# Targeting Lung Cancer Stem Cells with Antipsychological Drug Thioridazine

**DOI:** 10.1155/2016/6709828

**Published:** 2016-07-31

**Authors:** Haiying Yue, Dongning Huang, Li Qin, Zhiyong Zheng, Li Hua, Guodong Wang, Jian Huang, Haixin Huang

**Affiliations:** Department of Oncology, Liuzhou City Workers Hospital, The Fourth Affiliated Hospital of Guangxi Medical University, Liuzhou 545000, China

## Abstract

Lung cancer stem cells are a subpopulation of cells critical for lung cancer progression, metastasis, and drug resistance. Thioridazine, a classical neurological drug, has been reported with anticancer ability. However, whether thioridazine could inhibit lung cancer stem cells has never been studied. In our current work, we used different dosage of thioridazine to test its effect on lung cancer stem cells sphere formation. The response of lung cancer stem cells to chemotherapy drug with thioridazine treatment was measured. The cell cycle distribution of lung cancer stem cells after thioridazine treatment was detected. The in vivo inhibitory effect of thioridazine was also measured. We found that thioridazine could dramatically inhibit sphere formation of lung cancer stem cells. It sensitized the LCSCs to chemotherapeutic drugs 5-FU and cisplatin. Thioridazine altered the cell cycle distribution of LCSCs and decreased the proportion of G0 phase cells in lung cancer stem cells. Thioridazine inhibited lung cancer stem cells initiated tumors growth in vivo. This study showed that thioridazine could inhibit lung cancer stem cells in vitro and in vivo. It provides a potential drug for lung cancer therapy through targeting lung cancer stem cells.

## 1. Introduction

Lung cancer is the most common cancer in the world. It leads to a lot of patients dying of cancer every year. More than 1 million lung cancer patients died in 2012. The median five-year survival rate of lung cancer in the United States is about 16.8%. However, this percentage is even lower in developing countries. Lung cancer majorly consists of small-cell lung carcinoma and non-small-cell lung carcinoma (NSCLC). The majority, around 85%, of the lung cancer patients have been exposed to tobacco for a long time. Current treatment methods for lung cancer are mostly traditional methods, including surgery, chemotherapy, and radiotherapy. However, the resistance to chemo- or radiotherapy is a big issue for lung cancer therapy [1–3].

In the recent years, people found that there is a small subpopulation of cells in tumors, which play a key role in the resistance of cancer to chemotherapy and radiotherapy. In addition, they are also responsible for tumor progression and metastasis. They are named cancer stem cells or cancer initiating cells [4]. Leukemia stem cells are the first reported kind of cancer stem cells [5]. In 2003, researchers disclosed that very few CD44+CD24− cells could initiate tumors in mice. These populations of cells possess stem cell properties. This is the first work on cancer stem cells in solid tumor [6]. After that, cancer stem cells were reported in various kinds of cancers, including lung cancer, gastric cancer, brain cancer, liver cancer, and colon cancer [7–11].

After the discovery of cancer stem cells, researchers tried to look for efficient approaches to target cancer stem cells. Some groups screened the anticancer stem cells drug through small molecule screen. In 2012, through known molecule libraries screen, Sachlos et al. found that the classic antipsychotic drug, thioridazine, showed great anticancer stem cells ability [12]. After this report, people found that thioridazine possesses antitumor effect in several types of tumors [13–15]. However, till now, no one has reported whether thioridazine could target lung cancer stem cells. In this work, we tested the effect of thioridazine on LCSCs sphere formation, chemoresistance, cell cycle, and in vivo prohibitory function. Our work disclosed a novel function of thioridazine in LCSCs. This suggested thioridazine as an efficient drug for lung cancer therapy by targeting LCSCs.

## 2. Materials and Methods

### 2.1. Cells and Reagents

The NCI-H1299 and 95-D cells were purchased from Shanghai Cell Bank (Shanghai, China). Both cells were cultured in Dulbecco's Modified Eagle's Medium (DMEM) with 10% fetal bovine serum (FBS) supplemented with penicillin and streptomycin. The cells were maintained in incubator at 37°C and 5% CO_2_.

### 2.2. Sphere Formation

The lung cancer stem cells were accumulated by sphere formation. The NCI-H1299 cells and 95-D cells were trypsinized into single cells and washed with PBS twice. The cells were suspended in neuroblast medium with 20 ng/mL EGF, 20 ng/mL bFGF, and B27. The cells were cultured in ultralow attachment dishes for 7 days to form spheres. The spheres were centrifuged and trypsinized into single cells for further studies.

### 2.3. Cell Viability Assay

The NCI-H1299 cells and 95-D cells were seeded into 96-well dishes at 5000 cells/well. The cells were treated with different dosage of thioridazine and cell viability was measured 2 days later or cells were treated with 10 *μ*M thioridazine for indicated days. The cell viability was measured by MTT assay purchased from Beyotime, following the manufacturer's protocol.

### 2.4. Cell Cycle Assay

The LCSCs were treated with 0, 5, and 10 *μ*M thioridazine for 24 hrs. The cells were centrifuged and trypsinized into single cells. The cells were washed with PBS and fixed with 70% ethanol at 4°C for 30 min. The cells were centrifuged at 2000 rpm for 5 min and then washed with PBS thrice. After that, the cells were incubated with 50 *μ*g/mL RNAase at 37°C for 30 min and another 30 min with 50 *μ*g/mL propidium iodide in dark environment at room temperature. The data were collected on Calibur and analyzed by FlowJo.

### 2.5. Western Blot Assay

The western blot experiment was carried out following the standard protocol. The rabbit anti-GAPDH, pAkt, and Akt were purchased from Cell Signaling Technology. The secondary antibody goat anti-rabbit was obtained from Beyotime.

### 2.6. Animal Experiments

All the animal experiments were approved by the Institutional Review Board and conducted in accordance with the Declaration of Helsinki (1964). The 5-week-old nude mice were purchased from Shanghai SLAC company. The mice were kept in SPF environment. The NCI-H1299 LCSCs were pretreated with DMSO or 5 *μ*M thioridazine for 12 hrs. The cells were trypsinized into single cells and injected into the left and right back of nude mice, respectively. Each mouse was injected with 1 × 10^6^ cells/side. The size of the tumors was monitored and measured every 3 days. The tumor size was length × width × width/2.

## 3. Results

### 3.1. Thioridazine Inhibited Lung Cancer Cells in a Time and Dose Dependent Manner

**Figure 1:**
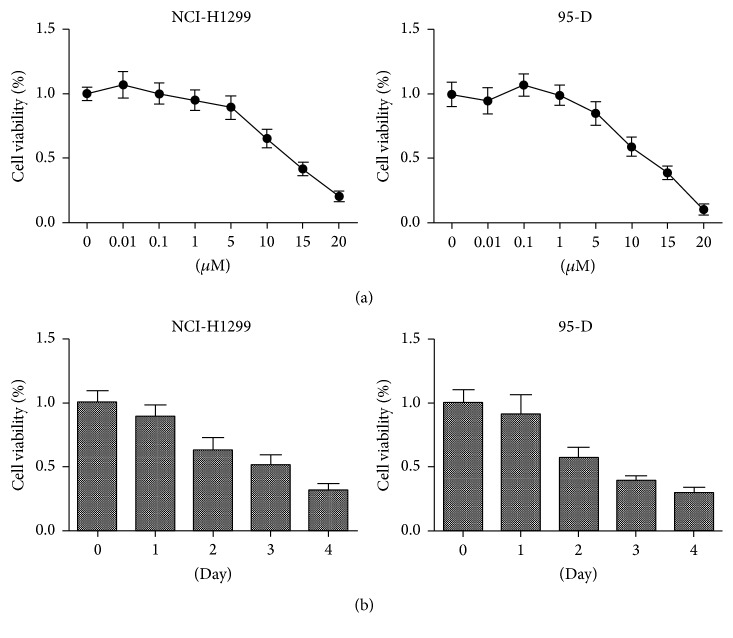
Thioridazine inhibited lung cancer cells in a time and dose dependent manner. (a) The NCI-H1299 and 95-D cells were treated with thioridazine at indicated dosage for 48 hrs. (b) The NCI-H1299 and 95-D cells were treated with 10 *μ*M thioridazine for different days.

To test whether thioridazine could efficiently prohibit lung cancer cells death, we used different dosage of thioridazine to treat lung cancer cells, NCI-H1299 cells and 95-D cells, for 2 days. Thioridazine robustly inhibited lung cancer cells growth in a dose dependent manner (Figure 1(a)). 10 *μ*M thioridazine treatment for indicated days showed that thioridazine prohibited NCI-H1299 cells and 95-D cells growth in a time dependent manner as well (Figure 1(b)).

### 3.2. Thioridazine Inhibited LCSCs Sphere Formation

**Figure 2:**
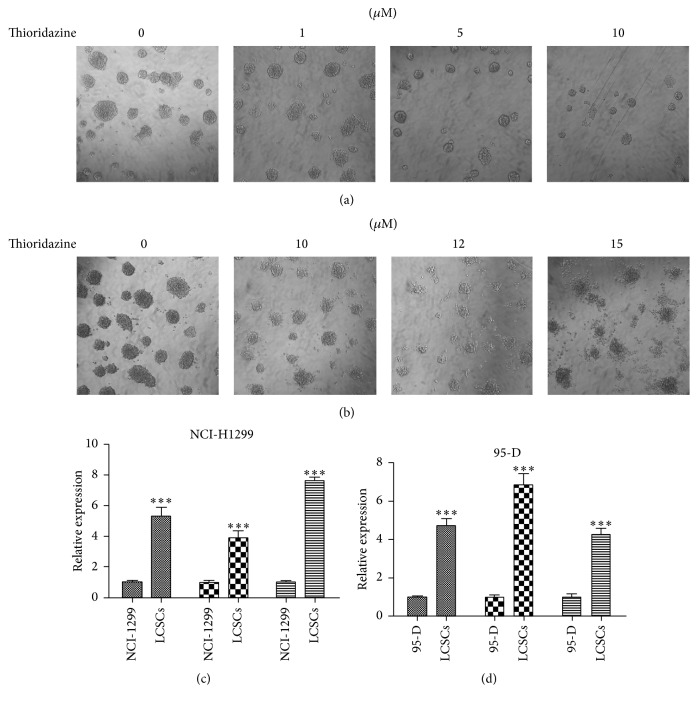
Thioridazine inhibited LCSCs sphere formation. (a) Low dosage thioridazine affected the sphere formation of NCI-H1299 stem cells. (b) High dosage thioridazine induced NCI-H1299 stem cells death. (c) The expression of stemness genes in NCI-H1299 sphere cells. (d) The expression of stemness genes in 95-D sphere cells. ∗∗∗ means *P* < 0.001.

To measure whether thioridazine inhibits lung cancer cells growth by affecting their cancer stem cells, we used specific medium to accumulate lung cancer stem cells in spheres and called them LCSCs. We detected the sphere formation efficiency of NCI-H1299 cells after thioridazine treatment. The spheres were cultured for 8 days. It was disclosed that thioridazine inhibited sphere formation of NCI-H1299 cells (Figure 2(a)). For higher dosage of thioridazine treatment, it not only inhibited the sphere formation of NCI-H1299 cells, but also induced cell death of NCI-H1299 stem cells (Figure 2(b)). To test whether the formed spheres are cancer stem cells, we detected the expression of stemness genes, Oct-4, Sox-2, and Nanog, in NCI-H1299 and 95-D sphere cells and regularly cultured cells. We found that the stemness genes were highly expressed in the sphere cells (Figures 2(c) and 2(d)).

### 3.3. Thioridazine Promoted LCSCs Sensitized to Chemotherapy

**Figure 3:**
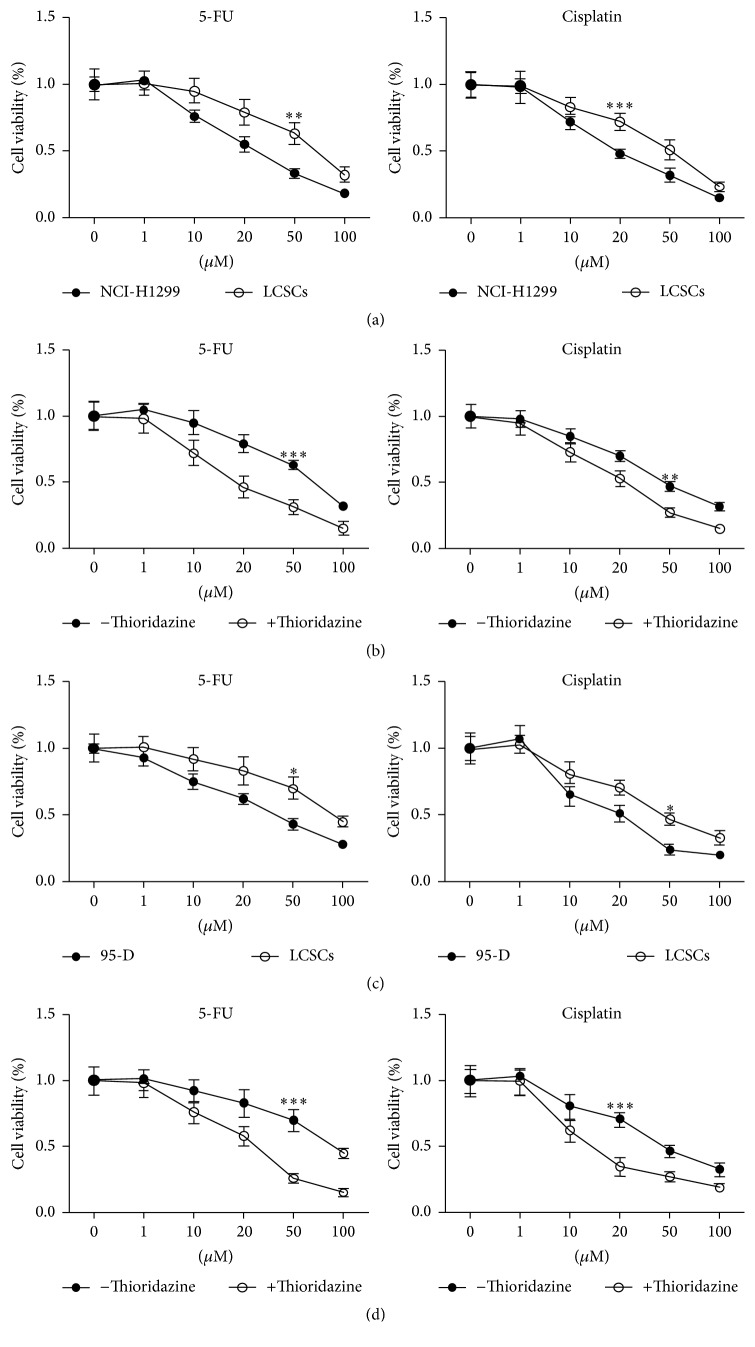
Thioridazine promoted LCSCs sensitized to chemotherapy. (a) NCI-H1299 sphere cells resisted chemotherapy drugs. (b) 10 *μ*M thioridazine sensitized NCI-H1299 stem cells to chemotherapy. (c) 95-D sphere cells resisted chemotherapy drugs. (d) 10 *μ*M thioridazine sensitized 95-D stem cells to chemotherapy. ^*∗*^ *P* < 0.05, ^*∗∗*^ *P* < 0.01, and ^*∗∗∗*^ *P* < 0.001.

One of the major points of cancer stem cells is their chemoresistance. The NCI-H1299 stem cells accumulated by sphere formation dramatically resisted 5-FU and cisplatin (Figure 3(a)). To check whether thioridazine could sensitize LCSCs to therapeutic drugs, the LCSCs were treated with chemotherapy drugs together with 10 *μ*M of thioridazine. Combined with thioridazine treatment, LCSCs were robustly sensitized to 5-FU and cisplatin (Figure 3(b)). This effect was further confirmed in 95-D cell derived cancer stem cells (Figures 3(c) and 3(d)).

### 3.4. Thioridazine Altered the Cell Cycle Distribution of LCSCs

**Figure 4:**
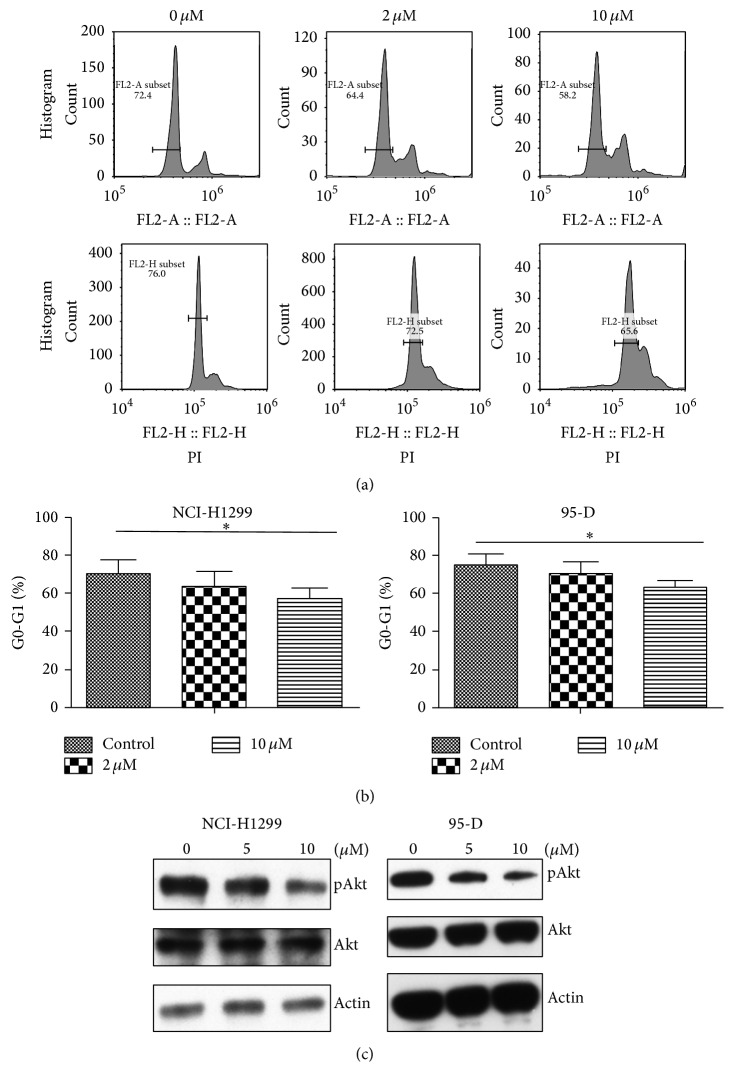
Thioridazine altered the cell cycle distribution of LCSCs. (a) Thioridazine altered the cell cycle distribution of NCI-H1299 stem cells (upper panel) and 95-D stem cells (lower panel). (b) Quantification of the proportion of G0/G1 phase cells in NCI-H1299 stem cells (left panel) and 95-D stem cells (right panel). (c) pAkt and Akt expression of NCI-H1299 stem cells (left panel) and 95-D stem cells (right panel) after thioridazine treatment. ^*∗*^ *P* < 0.05.

Cancer stem cells are mostly at quiescent status and thus resistant to chemotherapy. To test whether thioridazine altered the quiescence of LCSCs, we measured the cell cycle distribution of LCSCs. Thioridazine strongly decreased the proportion of G0/G1 phase cells (Figures 4(a) and 4(b)). Thioridazine can also decrease the phosphorylation of Akt, a critical gene related to survival and stemness (Figure 4(c)).

### 3.5. Thioridazine Prevented the LCSCs Initiated Tumors Growth

**Figure 5:**
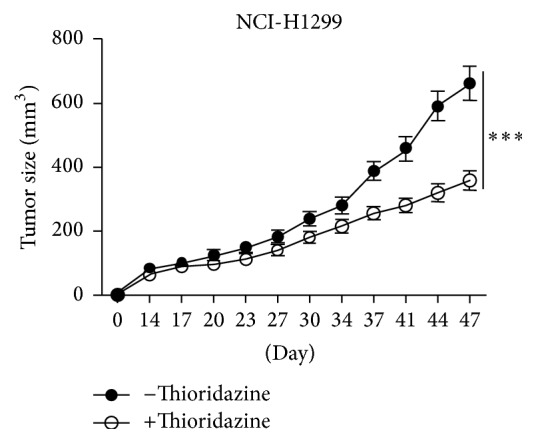
Thioridazine prevented the LCSCs initiated tumors growth. ^*∗∗∗*^ *P* < 0.001.

We also checked the inhibition of LCSCs initiated tumors growth in vivo. LCSCs pretreated with thioridazine for 12 hours showed decreased proliferation rate. They grew slower than those initiated by LCSCs with DMSO treatment (Figure 5). This result suggested that thioridazine prohibited tumor growth in vivo by targeting LCSCs.

## 4. Discussion

Lung cancer is the most common cancer in the world. It leads to the death of plenty of patients every year [1]. Currently, traditional methods for lung cancer therapy work but are confronted with the problem of cancer resistance. Looking for an effective way is urgent for lung cancer therapy. When the cancer stem cells theory was disclosed, people found that these small populations of cells played lots of critical roles in cancer. Researchers are looking for an effective and safe way to target the cancer stem cells.

At the current time, the major ways for cancer stem cells targeting are self-renewal inhibition and targeting with oncolytic viruses or through small molecular drug screen. The stemness associated pathways, Hedgehog, Notch, and Wnt, were considered as targets [16–18]. Targeting these pathways will make the cancer stem cells unable to self-renew and lead to tumor shrinking in the long run. Oncolytic adenovirus, herpes simplex virus, and reovirus could replicate in cancer stem cells and directly lyzed them or promoted their death [19–21]. The method of drug screen to look for an effective candidate in targeting cancer stem cells can obtain exciting molecules [12].

Thioridazine classically functions as an antipsychotic drug and was discovered many years ago. It has also been reported in cancer several years ago. However, its anticancer function has rarely been discovered until 2012. Researchers used drug screen approach to look for efficient drug for targeting cancer stem cells. The old drug thioridazine was discovered by this work. It showed strong anticancer stem cells property [12]. After that, the anticancer ability of thioridazine was reported in several kinds of cancers, including lung cancer [13–15]. Cancer stem cells are a critical subpopulation in lung cancer. They will result in cancer relapse after treatment. Eliminating lung cancer stem cells is critical for lung cancer therapy. However, whether thioridazine could target lung cancer stem cells has never been reported by any work before.

In this study, we firstly tested the ability of thioridazine to target lung cancer cells. It showed strong inhibiting efficiency and similarity to the previous report (Figure 1). Then, we checked the effect of thioridazine on the sphere formation of lung cancer stem cells. Thioridazine inhibited NCI-H1299 cells forming spheres (Figure 2(a)). High dosage of thioridazine robustly induced cell death of formed NCI-H1299 spheres (Figure 2(b)). Thioridazine may not only decrease the stemness of lung cancer stem cells, but also induce these cells' death. With more than one cytotoxic effect, thioridazine finally showed a great effect against lung cancer stem cells. The cancer stem cell population is the major part of the reason why tumors resist chemotherapy. In our study, in comparison to NCI-H1299 cells, accumulated cancer stem cells are more resistant to chemodrugs 5-FU and cisplatin. Cotreatment of the cancer stem cells with thioridazine and 5-FU or cisplatin dramatically inhibited NCI-H1299 stem cells growth. Thioridazine sensitized lung cancer stem cells to chemotherapeutic drugs (Figure 3). Cancer stem cells are mostly quiescent cells and stay at G0 phase. This kind of cell is less proliferative and resistant to chemotherapy or radiation therapy. We treated the lung cancer stem cells with thioridazine. It decreased the proportion of G0/G1 subpopulation cells (Figures 4(a) and 4(b)). Thioridazine also decreased the phosphorylation of Akt protein (Figure 4(c)). Akt is a critical protein for cell survival and it correlates with cancer stem cells as well. Decreasing Akt activity may lead to the inhibition of stemness of lung cancer stem cells. In addition to the in vitro effect of thioridazine, we found that thioridazine also inhibited NCI-H1299 stem cells initiated tumors growth (Figure 5).

Thioridazine showed a great effect against lung cancer stem cells in vitro and in vivo in our study. These results suggest thioridazine as an efficient drug for targeting lung cancer stem cells. It can eliminate the subpopulation of cancer stem cells in lung cancer. This will lead to long-term riddance of lung cancer with no tumor relapse. Moreover, thioridazine has already been approved by the FDA, making it easier to be used for cancer therapy. Thioridazine may be considered as a potential drug for lung cancer therapy.
